# Preoperative prognostic risk stratification model for papillary thyroid carcinoma based on clinical and ultrasound characteristics

**DOI:** 10.3389/fendo.2022.1025739

**Published:** 2022-10-07

**Authors:** Keyu Shen, Siqi Xiao, Xianji Wu, Guang Zhang

**Affiliations:** Department of Thyroid Surgery, The China-Japan Union Hospital of Jilin University, Jilin Provincial Key Laboratory of Surgical Translational Medicine, Jilin Provincial Precision Medicine Laboratory of Molecular Biology and Translational Medicine on Differentiated Thyroid Carcinoma, Changchun, China

**Keywords:** papillary thyroid carcinoma, prognostic risk stratification, preoperative evaluation, nomogram, ultrasound characteristics

## Abstract

**Background:**

The preoperative risk stratification for patients with papillary thyroid carcinoma (PTC) plays a crucial role in guiding individualized treatment. We aim to construct a predictive model that aids in distinguishing between patients with low-risk and high-risk PTC based on preoperative clinical and ultrasound characteristics.

**Materials and methods:**

Patients who underwent open surgery and were diagnosed with PTC *via* a postoperative pathological report between January 2020 and December 2020 were retrospectively reviewed. Data including basic information, preoperative ultrasound characteristics, thyroid function, and postoperative pathology characteristics were obtained. Univariate logistic regression analysis and least absolute shrinkage and selection operator regression analysis were performed to screen candidate variables. Finally, the preoperative predictive model for PTC was established based on the results of the multivariate logistic regression analysis.

**Results:**

A total of 1,875 patients with PTC were enrolled. Eight variables (sex, age, number of foci, maximum tumor diameter on ultrasound, calcification, capsule, lymph node status on ultrasound, and thyroid peroxidase (TPO) antibody level) significantly associated with risk stratification were included in the predictive model. A nomogram was constructed for clinical utility. The model showed good discrimination, and the area under the curve was 0.777 [95% confidence interval (CI): 0.752–0.803] and 0.769 (95% CI: 0.729–0.809) in the training set and validation set, respectively. The calibration curve exhibited a rather good consistency with the perfect prediction. Furthermore, decision curve analysis and clinical impact curve showed that the model had good efficacy in predicting the prognostic risk of PTC.

**Conclusions:**

The nomogram model based on preoperative indicators for predicting the prognostic stratification of PTC showed a good predictive value. This could aid surgeons in deciding on individualized precision treatments.

## 1 Introduction

Since the 1970s, an increasing incidence of thyroid cancer has been reported globally (1). According to the national cancer monitoring report released by the National Cancer Center of China in 2022, thyroid cancer is currently the fastest growing cancer among women (2). Papillary thyroid carcinoma (PTC) is the most common type, accounting for 90% of all thyroid cancer cases. Majority of PTC cases have indolent characteristics and slow progression, which are usually associated with a favorable prognosis. However, some PTC cases exhibit aggressive characteristics (such as vascular invasion, extrathyroidal extension, lymph node metastasis, disease recurrence, or distant metastasis), which could lead to a poor clinical outcome (3–5).

Currently, surgical intervention is the recommended treatment option for PTC. However, alternative therapeutic options besides classic surgery, active surveillance (AS), and thermal ablation therapy are gradually being considered. In 1993, Kuma Hospital in Japan began to implement AS for patients with low-risk PTC whose tumor diameter was <1 cm; cumulative data demonstrated that AS could be used as a first-line management for low-risk PTC (6). Recently, ultrasound imaging-guided thermal ablation has been performed on thyroid cancer, which is less damaging, has a faster recovery, and is more aesthetically pleasing than invasive surgery. The clinical management of patients with PTC should be tailored to their specific condition according to the prognostic risk stratification (3). The 2015 American Thyroid Association (ATA) guidelines are generally used to predict disease-specific mortality (7), and the American Joint Committee on Cancer (AJCC)/TNM classification is used to predict the risk of disease recurrence or persistence (8). For differentiated thyroid cancer, the Age, Metastases, External infiltration, Size (AMES) system and the Metastases, Age, Completeness of resection, Invasion, Size (MACIS) system are used for predicting prognostic risk stratification (9). These systems are all dependent on postoperative pathology reports. In many cases, it is difficult for clinical practitioners to distinguish between low-risk and high-risk PTC patients preoperatively.

Thus, this study aims to screen the potential predictive factors for patients with low-risk PTC and construct a preoperative prediction model. Additionally, a nomogram is to be constructed, with the aim of providing a reference for surgeons to develop individualized precision treatments.

## 2 Materials and methods

### 2.1 Patients

**Figure 1:**
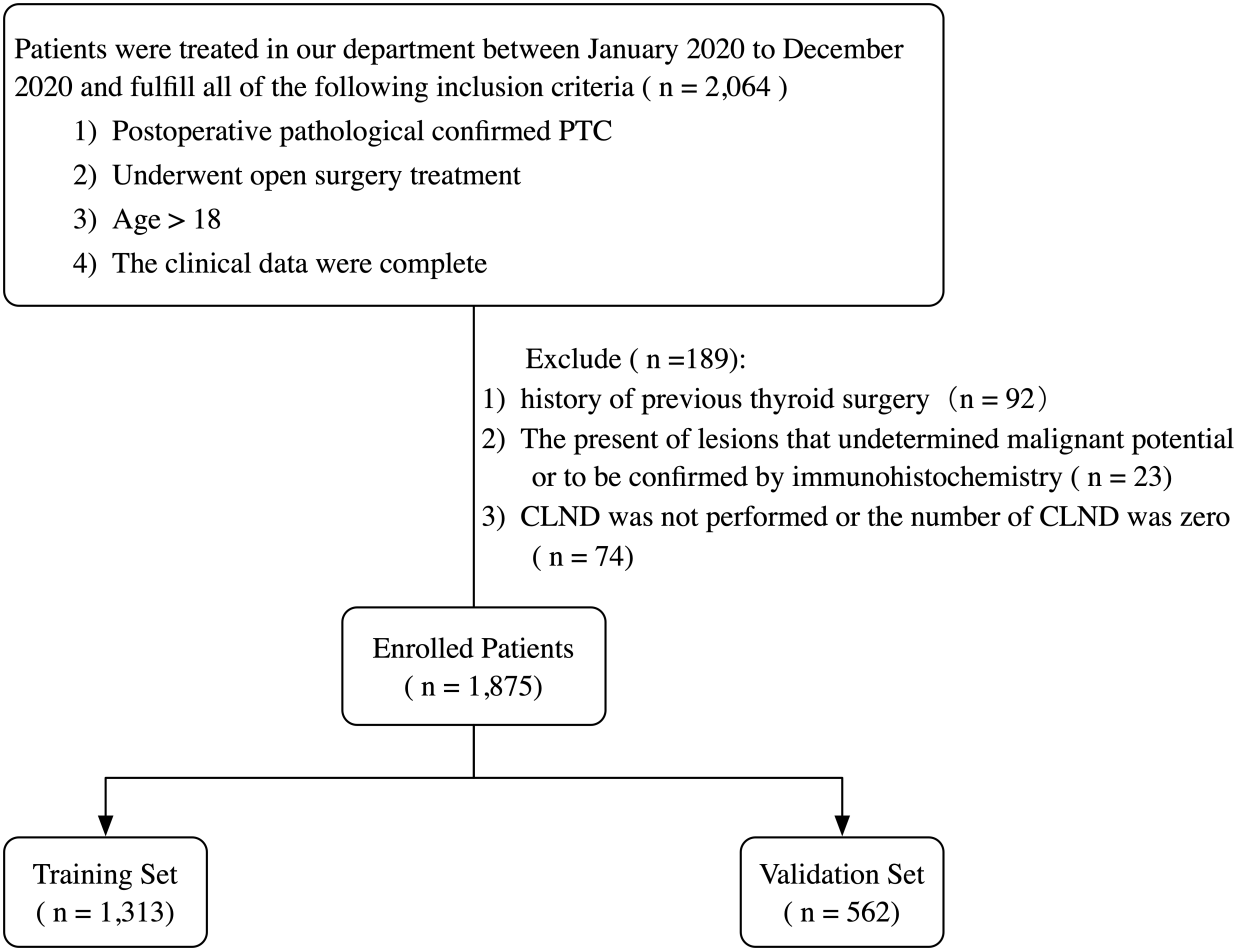
Flowchart of the patient selection process. PTC, papillary thyroid carcinoma; CLND, central lymph node dissection.

The records of patients who underwent open surgery and were diagnosed with PTC by postoperative pathology reports from January 2020 to December 2020 at the Department of Thyroid Surgery, China-Japan University, were retrospectively reviewed. All patients and their families voluntarily provided a written informed consent and agreed to participate in this study. The inclusion and exclusion criteria are summarized in **Figure 1**.

### 2.2 Surgical procedures

Ultrasound of the neck was performed in all patients before surgery to evaluate the primary lesions and determine the lymph node status. We performed unilateral thyroid lobectomy plus isthmectomy and ipsilateral central neck lymph node dissection for patients with unilateral PTC, while bilateral thyroid lobectomy and bilateral central neck lymph node dissection were performed for patients with bilateral PTC. Patients with preoperative imaging or fine-needle aspiration biopsy evidence of lateral lymph node metastasis underwent lateral neck dissection.

### 2.3 Risk stratification standard

We determined our prognostic risk stratification standard following the ATA guidelines, the AMES system, and the MACIS system. Patients with low-risk PTC must fulfill the following criteria: 1) maximum tumor diameter <5 cm; 2) no distant metastasis; 3) no microscopic invasion of tumor into the capsule or perithyroidal soft tissues; 4) clinical N0 or ≤5 pathologic N1 micrometastases (<0.2 cm in the largest dimension) (**Table 1**). Previous studies have demonstrated that based on these criteria, the structural recurrence risk of patients with low-risk PTC is approximately 5% and the 20-year survival rate is approximately 99%.

**Table 1:** Risk stratification standard of the present study.

Low-risk PTC	Middle–high-risk PTC
(with all of the following):	(with one of the following):
1) Maximum tumor diameter <5 cm	1) Maximum tumor diameter ≥5 cm
2) No distant metastasis	2) Distant metastasis
3) No microscopic invasion of tumor into the capsule or perithyroidal soft tissues	3) Microscopic invasion of tumor into the capsule or perithyroidal soft tissues
4) Clinical N0 or ≤5 pathologic N1 micrometastases (<0.2 cm in the largest dimension)	4) The largest dimension of metastatic lymph node ≥0.2 cm

PTC, papillary thyroid carcinoma.

### 2.4 Collected variables

The following information was reviewed and collected:

Basic clinical characteristics: sex (women and men), age, body mass index (BMI) (divided into two groups: <25 kg/m^2^ and ≥25 kg/m^2^), hypertension (yes or no), diabetes (yes or no), hyperthyroidism (yes or no).

Postoperative pathology report: pathological maximum tumor diameter, extrathyroidal extension (no extension, capsular invasion, extrathyroidal extension, perithyroidal soft tissue extension), number of metastatic lymph nodes, the largest dimension of the metastatic lymph node, distant metastasis (yes or no).

Preoperative ultrasound features of thyroid and lymph nodes: echogenicity of the thyroid gland (homogeneous, heterogeneous), location (upper, middle, lower), nodular echogenicity (hyperechoic or isoechoic, hypoechoic, marked hypoechoic), nodular composition (mixed cystic and solid, solid), number of foci, multifocality (unifocal, unilateral multifocal, bilateral multifocal), maximum tumor diameter on ultrasound (if tumors were multifocal, the size was determined using the largest foci), anteroposterior diameter/transverse diameter (A/T) (<1 and ≥1), shape (regular and irregular), margins (well-defined and ill-defined), calcification (absent, present), capsule (intact, tumor close to or invading the capsule), ultrasound-reported lymph node status (negative, suspicious).

Preoperative thyroid function test: TPO antibody level (reference: 0.00–0.34 pmol/L).

### 2.5 Statistical analysis

#### 2.5.1 Variable description and comparison

Statistical analysis was performed using R software (version 4.1.2). All statistical analyses were two-tailed, and *P* < 0.05 was considered statistically significant. The normality of continuous variable distributions was assessed using the Shapiro–Wilk normality test. Variables meeting the normal distribution were presented as mean ± standard deviation. Comparisons within groups were performed using a two-sample t-test. Variables that did not meet the normal distribution were presented as median, upper, and lower quartiles, and comparisons within groups were performed using the Mann–Whitney U test. Counting variables were expressed as constituent ratios (%) and analyzed by the chi-square test or Fisher’s exact probability test. Continuous variables were categorized using the cutoff values determined based on the receiver operating characteristic (ROC) curves.

#### 2.5.2 Model construction and assessment

Variables with *P* < 0.05 in the univariate logistic regression analysis were included in the least absolute shrinkage and selection operator (LASSO) regression analysis, which was used to identify potential predictive factors for risk stratification. Moreover, multivariate logistic regression analysis was performed to construct the predictive model. A nomogram was drawn to visualize the model. The possibility of low-risk stratification was quantified as a risk score according to the nomogram, and patients were divided into two subgroups (low risk and middle–high risk) using the cutoff point of the risk score. Grouping results of postoperative pathology and the preoperative predictive model were compared using the Fleiss’s Kappa test. ROC curves and calibration curves were used to evaluate the discrimination and calibration of our model. To evaluate the clinical significance, the decision curve analysis (DCA) and the clinical impact curve (CIC) were performed to calculate the net benefits at different threshold probabilities. The Box–Tidwell method was used to examine the assumption of linearity relationships between continuous independent variables and the logit transformed dependent variable. Furthermore, the variance inflation factor was used to analyze multicollinearity between independent variables. Data were detected for the presence of outliers, leverage points, and influential observations using Cook’s distance.

## 3 Results

### 3.1 Baseline clinical characteristics of enrolled patients

As summarized in **Table 2**, a total of 1,875 patients with PTC were enrolled in the study, including 400 men (21.3%) and 1,475 women (78.7%). The median age was 43 years; approximately half of the patients had a BMI ≥25 kg/m^2^ (43.5%); 201 patients (10.7%) had hypertension, 91 (4.9%) had diabetes, and 167 (8.9%) had hyperthyroidism. According to pathological reports, the median of the maximum tumor diameter was 0.6 cm; 167 (8.9%) patients had capsular invasion, 97 (5.2%) had an extrathyroidal extension, and 90 (4.8%) had perithyroidal soft tissue extension; the number of metastatic lymph nodes ranged from 0 to 34; the largest dimension of metastatic lymph node ranged from 0.0 to 5.5 cm; and six patients (0.3%) presented distant metastasis. Furthermore, preoperative ultrasound reports showed that 543 (29.0%) patients had a heterogeneous thyroid gland, with the largest half (53.0%) of the largest tumor located in the middle portion of the gland. Most of the tumors were hypoechoic (73.5%), and approximately all tumors (99.0%) were solid. Moreover, the number of foci ranged from 1 to 7, with 1,104 patients (58.9%) exhibiting solitary lesions, 281 (15.0%) displaying unilateral multifocality, and 490 (26.1%) exhibiting bilateral multifocality. The detailed description of the tumors by ultrasound is shown in **Table 2**. A total of 541 patients (28.9%) were suspected of having metastatic lymph nodes before surgery. The median preoperative TPO antibody level was 13.5 pmol/L.

**Table 2:** Baseline clinical characteristics of the enrolled patients.

	Total	Training Set	Validation Set	*P*-value
	(N = 1,875)	(N = 1,313)	(N = 562)
**Sex**				
Female	1,475 (78.7%)	1,043 (79.4%)	432 (76.9%)	0.237
Male	400 (21.3%)	270 (20.6%)	130 (23.1%)	
**Age (years)**				
Median [Q1, Q3]	43.0 [36.0, 50.0]	43.0 [35.0, 50.0]	43.0 [36.0, 50.0]	0.897
**BMI**				
<25 kg/m^2^	1,059 (56.5%)	738 (56.2%)	321 (57.1%)	0.754
≥25 kg/m^2^	816 (43.5%)	575 (43.8%)	241 (42.9%)	
**Hypertension**				
No	1,674 (89.3%)	1,171 (89.2%)	503 (89.5%)	0.903
Yes	201 (10.7%)	142 (10.8%)	59 (10.5%)	
**Diabetes**				
No	1,784 (95.1%)	1,255 (95.6%)	529 (94.1%)	0.220
Yes	91 (4.9%)	58 (4.4%)	33 (5.9%)	
**Hyperthyroidism**				
No	1,864 (99.4%)	1,305 (99.4%)	559 (99.5%)	1.000
Yes	11 (0.6%)	8 (0.6%)	3 (0.5%)	
**Pathological Maximum Tumor Diameter (cm)**				
Median [Q1, Q3]	0.600 [0.500, 1.00]	0.600 [0.400, 1.00]	0.600 [0.500, 1.00]	0.373
**Extrathyroidal Extension**				
No extension	1,521 (81.1%)	1,059 (80.7%)	462 (82.2%)	0.357
Capsular invasion	167 (8.9%)	119 (9.1%)	48 (8.5%)	
Extrathyroidal extension	97 (5.2%)	65 (5.0%)	32 (5.7%)	
Perithyroidal soft tissue extension	90 (4.8%)	70 (5.3%)	20 (3.6%)	
**Largest Dimension of the Metastatic Lymph Node (cm)**				
Median [Q1, Q3]	0 [0, 0.200]	0 [0, 0.200]	0 [0, 0.200]	0.194
**Number of Metastatic Lymph Node**				
Median [Q1, Q3]	0 [0, 2.00]	0 [0, 2.00]	0 [0, 2.00]	0.230
**Distant Metastasis**				
No	1,869 (99.7%)	1,309 (99.7%)	560 (99.6%)	1.000
Yes	6 (0.3%)	4 (0.3%)	2 (0.4%)	
**The Echogenicity of the Thyroid Gland**				
Homogeneous	1,332 (71.0%)	931 (70.9%)	401 (71.4%)	0.889
Heterogeneous	543 (29.0%)	382 (29.1%)	161 (28.6%)	
**Location**				
Upper	396 (21.1%)	284 (21.6%)	112 (19.9%)	0.254
Middle	993 (53.0%)	679 (51.7%)	314 (55.9%)	
Lower	486 (25.9%)	350 (26.7%)	136 (24.2%)	
**Nodular Echogenicity**				
Hyperechoic or isoechoic	18 (1.0%)	11 (0.8%)	7 (1.2%)	0.503
Hypoechoic	1,378 (73.5%)	959 (73.0%)	419 (74.6%)	
Marked hypoechoic	479 (25.5%)	343 (26.1%)	136 (24.2%)	
**Nodular Composition**				
Mixed cystic and solid	19 (1.0%)	13 (1.0%)	6 (1.1%)	1.000
Solid	1,856 (99.0%)	1,300 (99.0%)	556 (98.9%)	
**Number of foci**				
Median [Q1, Q3]	1.00 [1.00, 2.00]	1.00 [1.00, 2.00]	1.00 [1.00, 2.00]	0.107
**Multifocality**				
Unifocal	1,104 (58.9%)	787 (59.9%)	317 (56.4%)	0.314
Unilateral multifocal	281 (15.0%)	195 (14.9%)	86 (15.3%)	
Bilateral multifocal	490 (26.1%)	331 (25.2%)	159 (28.3%)	
**Maximum Tumor Diameter on Ultrasound (cm)**				
Median [Q1, Q3]	0.770 [0.580, 1.09]	0.770 [0.570, 1.09]	0.790 [0.603, 1.09]	0.428
**A/T**				
<1	842 (44.9%)	584 (44.5%)	258 (45.9%)	0.604
≥1	1,033 (55.1%)	729 (55.5%)	304 (54.1%)	
**Shape**				
Regular	1,051 (56.1%)	739 (56.3%)	312 (55.5%)	0.798
Irregular	824 (43.9%)	574 (43.7%)	250 (44.5%)	
**Margins**				
Well-defined	605 (32.3%)	424 (32.3%)	181 (32.2%)	1.000
Ill-defined	1,270 (67.7%)	889 (67.7%)	381 (67.8%)	
**Calcification**				
Absent	852 (45.4%)	591 (45.0%)	261 (46.4%)	0.604
Present	1,023 (54.6%)	722 (55.0%)	301 (53.6%)	
**Capsule**				
Intact	1,639 (87.4%)	1,147 (87.4%)	492 (87.5%)	0.971
Tumors close to or invading	236 (12.6%)	166 (12.6%)	70 (12.5%)	
**Ultrasound-Reported Lymph Node Status**				
Negative	1,334 (71.1%)	951 (72.4%)	383 (68.1%)	0.069
Suspicious	541 (28.9%)	362 (27.6%)	179 (31.9%)	
**TPO Antibody Level (pmol/L)**				
Median [Q1, Q3]	13.5 [9.40, 22.6]	13.4 [9.30, 21.6]	13.8 [9.73, 23.6]	0.176

BMI, body mass index; A/T, anteroposterior diameter/ transverse diameter; TPO, thyroid peroxidase.

Finally, 1,875 patients were randomly divided into the training (n = 1,313) and validation (n = 562) sets in a 7:3 ratio. Notably, no significant difference in clinicopathological characteristics and ultrasound features was observed between the two groups (*P* > 0.05).

### 3.2 Logistic and least absolute shrinkage and selection operator (LASSO) regression analysis in the training set

**Figure 2:**
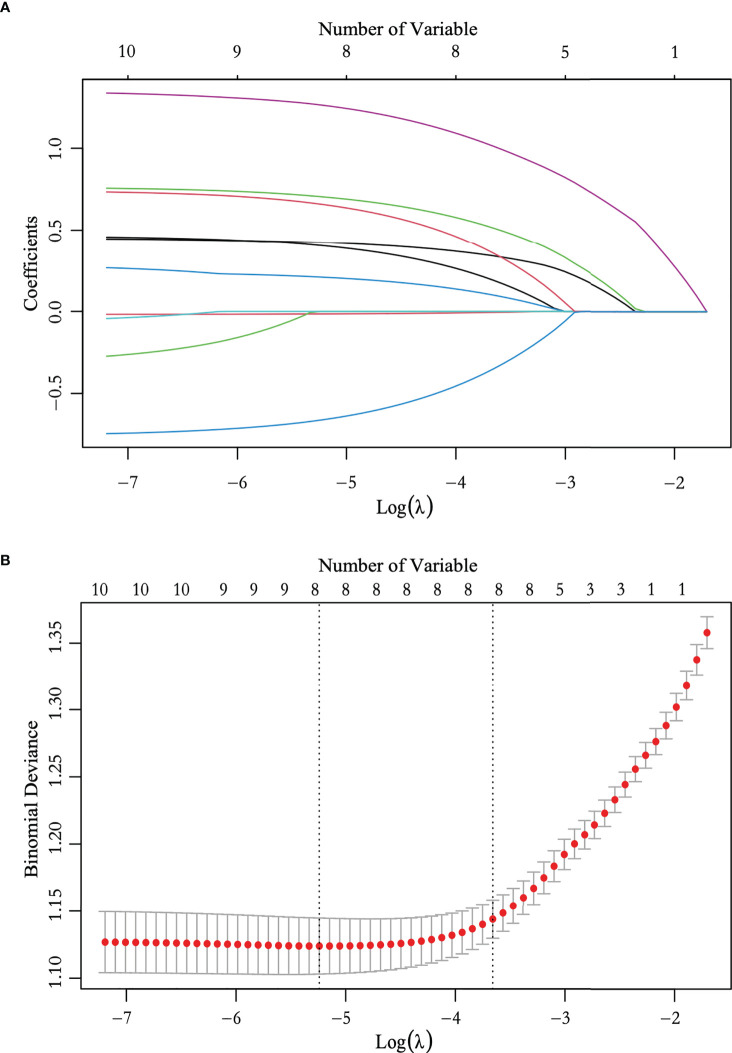
Feature selection using the least absolute shrinkage and selection operator (LASSO) logistic regression. **(A)** LASSO coefficient profile plots of the 10 variables. **(B)** The selection of optimal predictive variables using cross-validation. Dotted vertical lines were drawn at the optimal values using the minimum criteria and the one-fold standard error of the minimum criteria.

We performed ROC curve analysis and identified 26.450 pmol/L as the best cutoff value of TPO antibody level, with 83.5% sensitivity, 24.7% specificity, and 0.545 area under curve (AUC). Univariate analysis revealed significant differences in sex, age, nodular composition, number of foci, multifocality, maximum tumor diameter on ultrasound, calcification, capsule, ultrasound-reported lymph node status, and TPO antibody level (*P* < 0.05) (**Table 3**). These variables were included in the LASSO regression analysis, which revealed eight predictive factors (with non-zero coefficients) for patients with low-risk PTC, including sex, age, number of foci, maximum tumor diameter on ultrasound, calcification, capsule, ultrasound-reported lymph node status, and TPO antibody level (**Figure 2**).

**Table 3:** Univariate analysis of the predictive factors.

Variable		Univariate Logistic Regression			
		OR	CI	*P*	
**Sex**	Women vs. Men	0.55	0.42-0.73	<0.001	***
**Age**		1.03	1.02-1.04	<0.001	***
**BMI**	<25 vs. ≥25 kg/m^2^	0.93	0.74-1.16	0.51	
**Hypertension**	No vs. yes	1.30	0.90-1.86	0.16	
**Diabetes**	No vs. yes	1.26	0.73-2.18	0.41	
**Hyperthyroidism**	No vs. yes	2.13	0.43-10.60	0.36	
**The Echogenicity of the Thyroid Gland**	Homogeneous vs. Heterogeneous	1.28	1.01-1.64	0.05	
**Location**	Upper vs. Lower	1.11	0.84-1.46	0.48	
	Upper vs. Middle	1.22	0.89-1.68	0.22	
**Nodular Echogenicity**	Hyperechoic/isoechoic vs. Marked hypoechoic	2.38	0.69-8.20	0.17	
	Hyperechoic/isoechoic vs. Hypoechoic	2.83	0.81-9.86	0.10	
**Nodular Composition**	Cystic and Solid vs. Solid	4.78	1.31-17.46	0.02	**
**Number of foci**		0.78	0.70-0.87	<0.001	***
**Multifocality**	Unifocal vs. Unilateral multifocal	0.76	0.55-1.04	0.09	
	Unifocal vs. Bilateral multifocal	0.62	0.48-0.80	<0.001	***
**Maximum Tumor Diameter on Ultrasound**		0.20	0.15-0.26	<0.001	***
**A/T**	<1 vs. ≥1	1.14	0.91-1.42	0.26	
**Shape**	Regular vs. Irregular	0.85	0.68-1.05	0.14	
**Margins**	Well-defined vs. Ill-defined	1.19	0.94-1.51	0.14	
**Calcification**	Absent vs. Present	0.35	0.28-0.44	<0.001	***
**Capsule**	Intact vs. Close to or invading	0.44	0.32-0.61	<0.001	***
**Ultrasound-Reported Lymph Node Status**	Negative vs. Suspicious	0.34	0.26-0.44	<0.001	***
**TPO Antibody Level**	<26.45 vs. ≥26.45 pmol/L	1.66	1.25-2.19	<0.001	***

BMI, body mass index; A/T, anteroposterior diameter/ transverse diameter; TPO, thyroid peroxidase. Significance: ^∗∗^P < 0.05; ^∗∗∗^P < 0.01.

Multivariate analysis was performed to develop the predictive model, and the results showed that men [odds ratio (OR): 2.992, 95% confidence interval (CI): 0.162–0.775, *P* = 0.003], age (OR: -2.776, 95% CI: -0.031 to -0.005, *P* = 0.006), number of foci (OR: 3.901, 95% CI: 0.121–0.365, *P* < 0.001), maximum tumor diameter on ultrasound (OR: 9.153, 95% CI: 1.062–1.640, *P* < 0.001), calcification presence (OR: 3.290, 95% CI: 0.181–0.714, *P* = 0.001), tumor close to or invading the capsule (OR: 3.890, 95% CI: 0.372–1.128, *P* < 0.001), the existence of suspicious lymph nodes (OR: 5.189, 95% CI: 0.476–1.055, *P* < 0.001), and TPO antibody level ≥26.45 pmol/L (OR: 3.901, 95% CI: 0.121–0.365, *P* < 0.001) remained independent predictive variables of low-risk PTC (**Table 4**).

**Table 4:** Multivariate analysis of the predictive factors.

Variable		Coefficient	Standard Error	OR	*P*-value	
**Sex**	Women vs. Men	0.468	0.156	2.992	0.003	**
**Age**		-0.018	0.007	-2.776	0.006	**
**Number of foci**		0.243	0.062	3.901	<0.001	***
**Maximum Tumor Diameter on Ultrasound**		1.351	0.148	9.153	<0.001	***
**Calcification**	Absent vs. Present	0.448	0.136	3.290	0.001	**
**Capsule**	Intact vs. Close to or invading	0.750	0.193	3.890	<0.001	***
**Ultrasound-Reported Lymph Node Status**	Negative vs. Suspicious	0.766	0.148	5.189	<0.001	***
**TPO Antibody Level**	<26.45 vs. ≥26.45 pmol/L	-0.760	0.167	-4.542	<0.001	***

TPO, thyroid peroxidase. Significance: ^∗∗^P < 0.05; ^∗∗∗^P < 0.01.

### 3.3 Construction and validation of the nomogram

**Figure 3:**
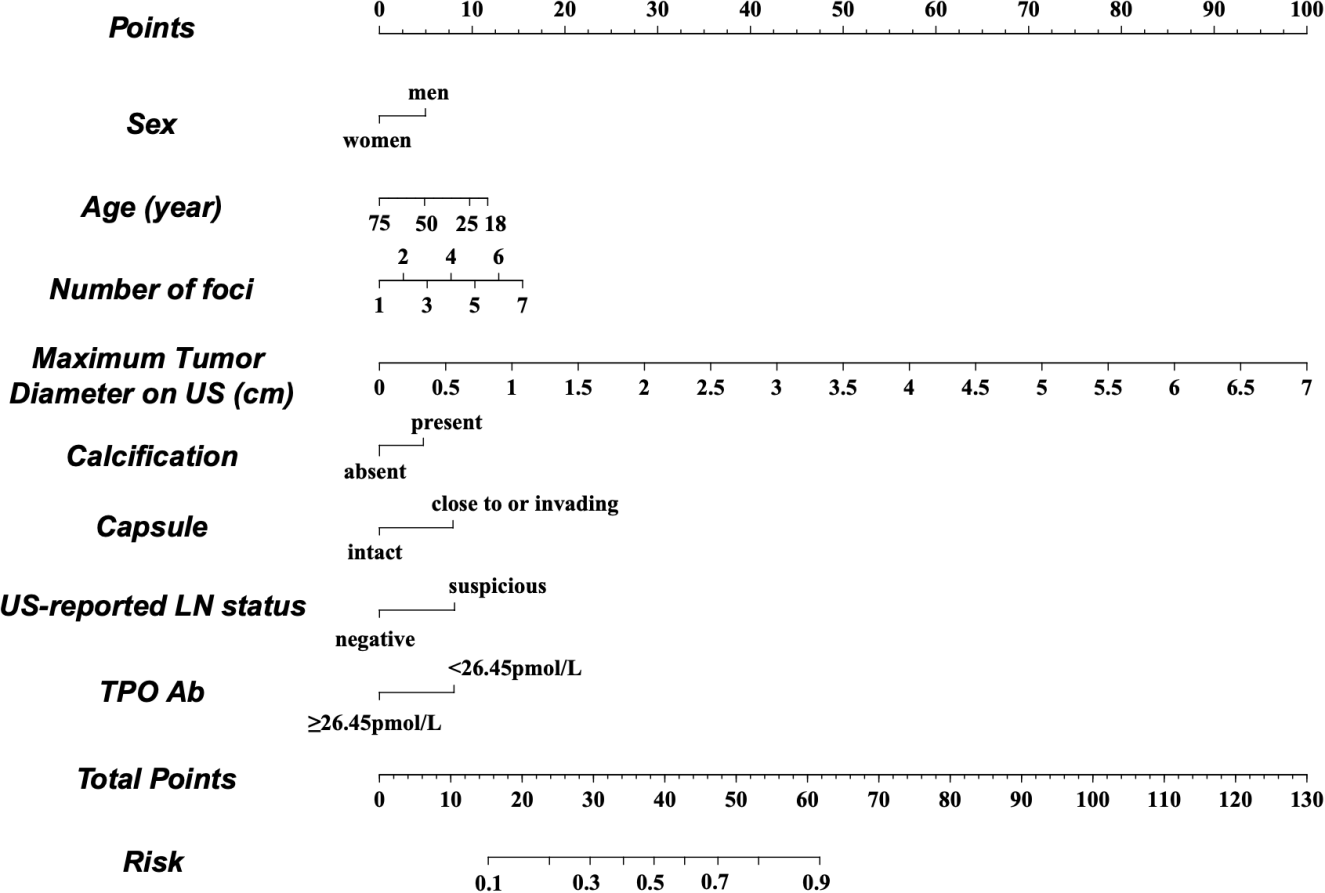
Preoperative factor-based nomogram used for predicting the prognostic risk in patients with PTC. US, ultrasound; LN, lymph node; TPO, thyroid peroxidase.

**Figure 4:**
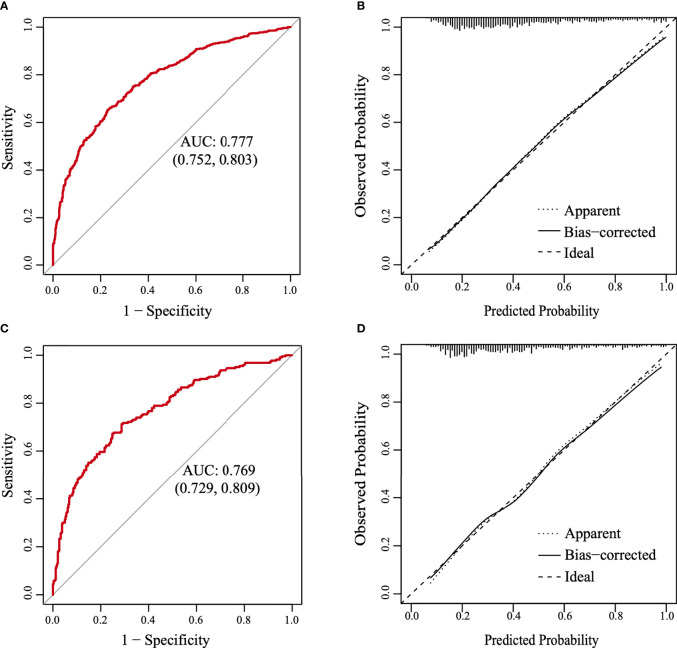
The receiver operating characteristic (ROC) curve and the calibration curve. **(A)** The ROC curve in the training set. **(B)** The calibration curve in the training set. **(C)** The ROC curve in the validation set. **(D)** The calibration curve in the validation set.

A nomogram was constructed based on the results of the multivariate analysis (**Figure 3**), with an optimistic C-index of 0.777 (**Figure 4A**). According to the best cutoff point of the risk score in the nomogram, patients were divided into a low-risk group (risk score <34.899) and middle–high-risk group (risk score ≥34.899). The Kappa test revealed that the risk stratification results of the preoperative prediction model and the postoperative pathological report had moderate consistency (Kappa value = 0.421, *P* < 0.001) (**Table 5**).

**Table 5:** Consistency check of the grouping results based on postoperative pathology and the preoperative predictive model.

Pathological Stratification	Preoperative Predictive Stratification		Total	κ	*P*-value
	Low risk	Middle–High risk		
**Low risk**	835 (76.7%)	273 (34.7%)	1,108 (59.1%)
**Middle–high risk**	254 (23.3%)	513 (65.3%)	767 (40.9%)	0.421	<0.001
**Total**	1,089 (58.1%)	786 (41.9%)	1,875

**Figure 5:**
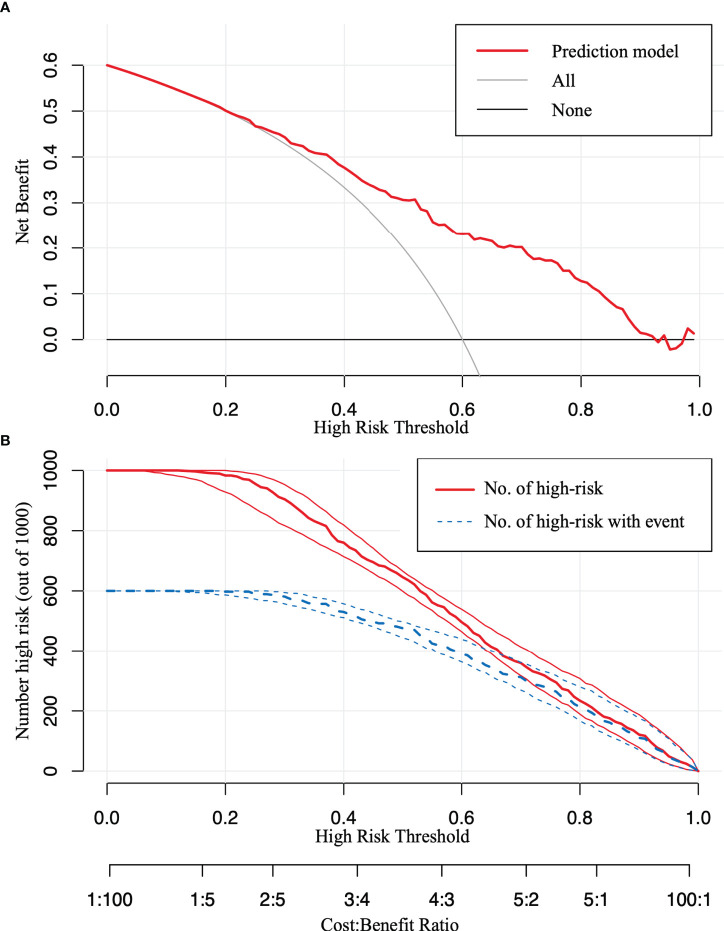
The decision curve analysis (DCA) and the clinical impact curve (CIC). **(A)** The DCA in the validation set. **(B)** The CIC in the validation set. The red curve [number of high-risk papillary thyroid carcinoma (PTC) individuals] indicates the number of people who are classified as positive (low-risk PTC) by the model at each threshold probability; the blue curve (number of high-risk PTC individuals with an outcome) represents the number of true positives at each threshold probability.

The model was further validated using the validation set with an AUC of 0.769 (95% CI: 0.729–0.809) (**Figure 4C**). Moreover, the Hosmer–Lemeshow test (training set: χ^2^ = 1.149, *P* = 0.563 > 0.05; validation set: χ^2^ = 3.126, *P* = 0.210 > 0.05) provided evidence of a favorable goodness of fit. Calibration curves were close to 45° in both sets (**Figures 4B, D** **)**. Furthermore, the DCA curve revealed that the nomogram risk stratification would have more net benefits than the treat-none or treat-all strategy when the threshold probability ranged from 20% to 95% (**Figure 5A**). The CIC (**Figure 5B**) was determined by predicting the risk stratification of 1,000 subjects using the bootstrap technique. The red curve (number of high-risk PTC individuals) indicates the number of people who are classified as positive (low-risk PTC) by the model at each threshold probability; the blue curve (number of high-risk PTC individuals with an outcome) represents the number of true positives at each threshold probability. The red curve was above the blue one in all threshold probabilities.

## 4 Discussion

With recent advancements in health awareness and the widespread use of ultrasound, the detection of PTC is increasing (10). However, the diagnosis and treatment of PTC remain controversial. Although the morbidity of PTC is high, mortality rates have remained at a low level (11). An increasing number of studies on the prognostic risk of PTC have modified the clinical practice from a “one-size-fits-all” management strategy to a precise individualized approach (1, 12). Domestic and international studies are currently actively exploring the best management strategy for patients with PTC. However, the lack of effective means to distinguish between patients with low-risk PTC and patients with middle–high-risk PTC in the early stage of disease diagnosis presents a major challenge. Therefore, we need a preoperative risk assessment system to identify the optimal treatment for each patient.

A review of recent studies on preoperative predictive models highlighted that current models focus on a single adverse outcome, such as central lymph node metastasis (CLNM) (13), extrathyroidal extension (14), or tumor recurrence/persistence (15). However, to the best of our knowledge, no studies have used prognostic risk stratification as an outcome to construct a prediction model. Moreover, the commonly used risk stratification systems (ATA guidelines and AJCC/TNM classification) are dependent on postoperative pathology reports, making it difficult to use them preoperatively.

In the present study, a total of 1,875 patients with PTC were enrolled. We used the training set to explore the potential prediction factors that impacted prognostic stratification. Among them, 769 of 1,313 cases (58.5%) were low risk, which was consistent with the fact that most patients with PTC have a good prognosis. Univariate logistic and LASSO regression analyses were performed to screen candidate variables. Consistent with previous findings, the male gender, the presence of suspicious lymph nodes, and an increase in tumor number and size were identified as risk factors of middle–high-risk PTC (the latter two were associated with tumor load) (16–18). Huang et al. (19) reported that microcalcification is an independent risk factor of CLNM. Moreover, the American College of Radiology Thyroid Imaging, Reporting and Data System (ACR-TIRADS) system also subdivided the calcification into macrocalcification, peripheral (rim) calcification, and microcalcification, which were assigned different values (20). Notably, no significant difference was observed between the different types of calcifications in prognostic stratification; therefore, we categorized them into one group. Nonetheless, tumors with calcifications revealed a higher prognostic risk than those without. Extrathyroidal invasion is often associated with poor outcomes. Bortz et al. (21) also indicated that all levels of extrathyroidal extension, including microscopic extension, were associated with an increased risk for nodal and distant metastasis. Similar conclusions can be drawn from our study. Meanwhile, we identified that older age and a higher level of TPO antibodies (≥26.45 pmol/L) were protective factors against poor prognosis. The probability of disease progression is higher in younger patients compared to older patients (22). The eighth edition of AJCC staging uses 55 years as a threshold for stratification (8). Similarly, we used the same standard for subgrouping; however, the predicting value of age disappeared. Therefore, we included age as a continuous variable in the model, with both univariate and multivariate analyses validating that age was significantly related to risk stratification. Thus, with increasing age, the prognostic risk decreased. PTC often co-occurs with thyroiditis; however, this relationship remains controversial (23, 24). Some studies have suggested that thyroiditis has a protective effect (25, 26); however, our results demonstrate that patients with higher TPO antibody levels preoperatively tend to have a lower risk of prognosis. Additionally, multivariate analysis revealed that the candidate variables screened using univariable analysis and LASSO analysis have been further proven to have a significant association with risk stratification.

Moreover, to visualize the model and facilitate broad clinical applications, a nomogram was constructed and the cutoff point was determined. The C-index was 0.777, illustrating that our model could significantly distinguish low-risk PTC from middle–high-risk PTC preoperatively. Moreover, our model showed moderate consistency based on the pathological stratification results. The model was further validated in the validation set and showed good accuracy. The Hosmer–Lemeshow test and the calibration curves indicated that our model had good consistency in actual situations. Furthermore, the DCA and the CIC visually revealed that the nomogram conferred a high clinical net benefit and confirmed the clinical applicability of our predictive model.

Nonetheless, there are limitations to this study. The present study was conducted as a single-center study, and thus, single-center effects cannot be excluded. External validation was not performed in additional datasets to evaluate the generalization capability of the model fairly. Therefore, multicenter and prospective studies are needed in the future.

## 5 Conclusion

This study shows that the male gender, younger age, increased tumor foci and size, presence of calcification and suspicious lymph nodes, tumors close to or invading the capsule, and low serum TPO antibody levels (<26.450 pmol/L) were significantly associated with the middle–high-risk stratification in patients with PTC. Furthermore, the constructed nomogram aids in predicting the prognostic risk preoperatively, which helps clinical practitioners select individualized treatment plans for each patient.

## Data availability statement

The original contributions presented in the study are included in the article/supplementary material. Further inquiries can be directed to the corresponding author.

## Author contributions

GZ and KS contributed to the study conception and design. Material preparation and data collection were carried out by KS, SX, and XW. All authors proposed many professional suggestions when data analysis. The first draft of the manuscript was written by KS. GZ contributed to manuscript review and editing. GZ was responsible for project administration and supervision. All authors contributed to the article and approved the submitted version.

## Funding

This study was supported by the Jilin Province educational program [JJKH20201055KJ]; the Program of Jilin Provincial Science and Technology Department, Basic Division [20200201580JC].

## Acknowledgments

The authors sincerely thank all the clinicians in the division for their support. They were Hui Sun, Nan Liang, and Tianyu Yu.

## Conflict of interest

The authors declare that the research was conducted in the absence of any commercial or financial relationships that could be construed as a potential conflict of interest.

## Publisher’s note

All claims expressed in this article are solely those of the authors and do not necessarily represent those of their affiliated organizations, or those of the publisher, the editors and the reviewers. Any product that may be evaluated in this article, or claim that may be made by its manufacturer, is not guaranteed or endorsed by the publisher.
